# Engineering tumor-oxygenated nanomaterials: advancing photodynamic therapy for cancer treatment

**DOI:** 10.3389/fbioe.2024.1383930

**Published:** 2024-03-13

**Authors:** Tingting Zuo, Xiaodie Li, Xuan Ma, Ye Zhang, Xueru Li, Xuehai Fan, Mingze Gao, Donglin Xia, Huijun Cheng

**Affiliations:** ^1^ College of Biological Sciences and Technology, Yili Normal University, Yining, China; ^2^ Department of Oncology, Zhujiang Hospital, Southern Medical University, Guangzhou, Guangdong, China; ^3^ No. 1 Traditional Chinese Medicine Hospital in Changde, Changde, China; ^4^ School of Public Health of Nantong University, Nantong, China; ^5^ Xinjiang Key Laboratory of Lavender Conservation and Utilization, Yining, China

**Keywords:** nanomaterials, photodynamic therapy, reactive oxygen species, hypoxia, tumor oxygenation

## Abstract

Photodynamic therapy (PDT), a promising treatment modality, employs photosensitizers to generate cytotoxic reactive oxygen species (ROS) within localized tumor regions. This technique involves administering a photosensitizer followed by light activation in the presence of oxygen (O_2_), resulting in cytotoxic ROS production. PDT’s spatiotemporal selectivity, minimally invasive nature, and compatibility with other treatment modalities make it a compelling therapeutic approach. However, hypoxic tumor microenvironment (TME) poses a significant challenge to conventional PDT. To overcome this hurdle, various strategies have been devised, including in-situ O_2_ generation, targeted O_2_ delivery, tumor vasculature normalization, modulation of mitochondrial respiration, and photocatalytic O_2_ generation. This review aims to provide a comprehensive overview of recent developments in designing tumor-oxygenated nanomaterials to enhance PDT efficacy. Furthermore, we delineate ongoing challenges and propose strategies to improve PDT’s clinical impact in cancer treatment.

## 1 Introduction

Photodynamic therapy (PDT) has garnered recognition due to its selective targeting and eradication of cancer cells while adopting a noninvasive approach (Correia et al., 2021; Shen et al., 2021; Siddhardha and Prasad, 2024). During PDT, the reaction between photosensitizers (PSs) and local oxygen produces reactive oxygen species (ROS), ultimately leading to tumor cell destruction (An et al., 2022). Alongside conventional PDT agents like indocyanine green and chlorin e6, substantial progress has been made in developing novel PDT agents, including metal-based, semiconductor-based, and composite oxide-based agents (Ge et al., 2014; Baskaran et al., 2018). These new agents demonstrate unique properties and exceptional potential, such as: 1) enabling enhanced photocatalytic activity, resulting in increased ROS production and improved tumor cell destruction (Prasad et al., 2017); 2) providing tunable absorption spectra and photophysical characteristics, facilitating personalized treatment approaches based on specific tumor types (Zhang et al., 2015; Lee et al., 2018); 3) facilitating easy modification for targeted drug delivery to tumor cells, enhancing treatment specificity while minimizing off-target effects (Sun et al., 2021; Hong et al., 2023); 4) offering substantial promise for personalized cancer therapy by enabling tailored treatment strategies according to individual patient characteristics. However, the majority of PDT agents generate ROS by reacting with oxygen within tumors, consequently exacerbating the hypoxic tumor microenvironment (TME) and significantly limiting both PDT efficacy and its clinical applicability (Jung et al., 2017; Sies and Jones, 2020). Additionally, the surviving tumor cells, being invulnerable to PDT, often develop increased resistance, further promoting tumor metastasis and recurrence. Therefore, overcoming tumor hypoxia could serve as a viable approach for boosting the anti-tumor efficacy of PDT treatment.

Hypoxia, characterized by oxygen (O_2_) deprivation, is a prevalent feature in many malignancies (Bristow and Hill, 2008; Fearon et al., 2016). This occurs due to the insufficient development of vasculature, which fails to meet the rapid proliferation demands of tumor cells. In attempts to address this issue, *in vitro* generation and transportation of O_2_ is efficiently controlled with some small molecules like perfluorooctyl bromide and perfluorotributylamine (Xu et al., 2016; Gonzalez et al., 2019; Zhi et al., 2023). However, their short blood half-life and limited tumor accessibility greatly restrict their effectiveness in oxygenating tumors *in vivo* (Hainfeld et al., 2018). Fortunately, the rapidly advancing field of nanotechnology has recently been employed to overcome the drawbacks associated with these O_2_-generating/delivering small molecules (Ashammakhi et al., 2020). This novel approach has successfully facilitated tumor oxygenation and enhanced the anti-tumor effects of PDT treatment. By utilizing nanotechnology, it is now possible to develop O_2_-carrying or O_2_-generating nanoparticles that can selectively accumulate in tumor tissues and effectively deliver O_2_ to the hypoxic regions (Liu et al., 2018). These advanced nanosystems exhibit several advantages compared to conventional small molecules (Milane et al., 2011). They have extended circulation times in the bloodstream, allowing for prolonged O_2_ delivery to the tumor site. Moreover, their small size and surface modifications enable efficient penetration into the TME, enhancing tumor accessibility and oxygenation. Additionally, these nanosystems can be engineered to possess targeting ligands that specifically recognize tumor cells, further improving their selectivity and efficacy (Sheng et al., 2017). Furthermore, nanostructured materials offer versatility in terms of customization for the controlled release of O_2_. By designing nanoparticle systems with precisely regulated properties, such as porosity, surface charge, and degradation rate, the release of O_2_ can be finely tuned to meet the specific demands of different tumor types and stages (Roy et al., 2018; Liang et al., 2023). This personalized approach holds great promise for maximizing the therapeutic effects of PDT while minimizing off-target effects and toxicity (Shen et al., 2021). Collectively, the integration of nanotechnology into the field of O_2_ delivery systems has revolutionized the approach to tackling tumor hypoxia (Wang H. et al., 2020; Lai et al., 2022). By exploiting the unique properties and capabilities of nanosystems, researchers are now able to overcome the limitations associated with small molecule-based strategies. This exciting development paves the way for improved tumor oxygenation and enhanced anti-tumor efficacy in PDT treatment, bringing us closer to more targeted and effective cancer therapies (Kumari et al., 2020; Sivasubramanian and Lo, 2022).

**SCHEME 1:**
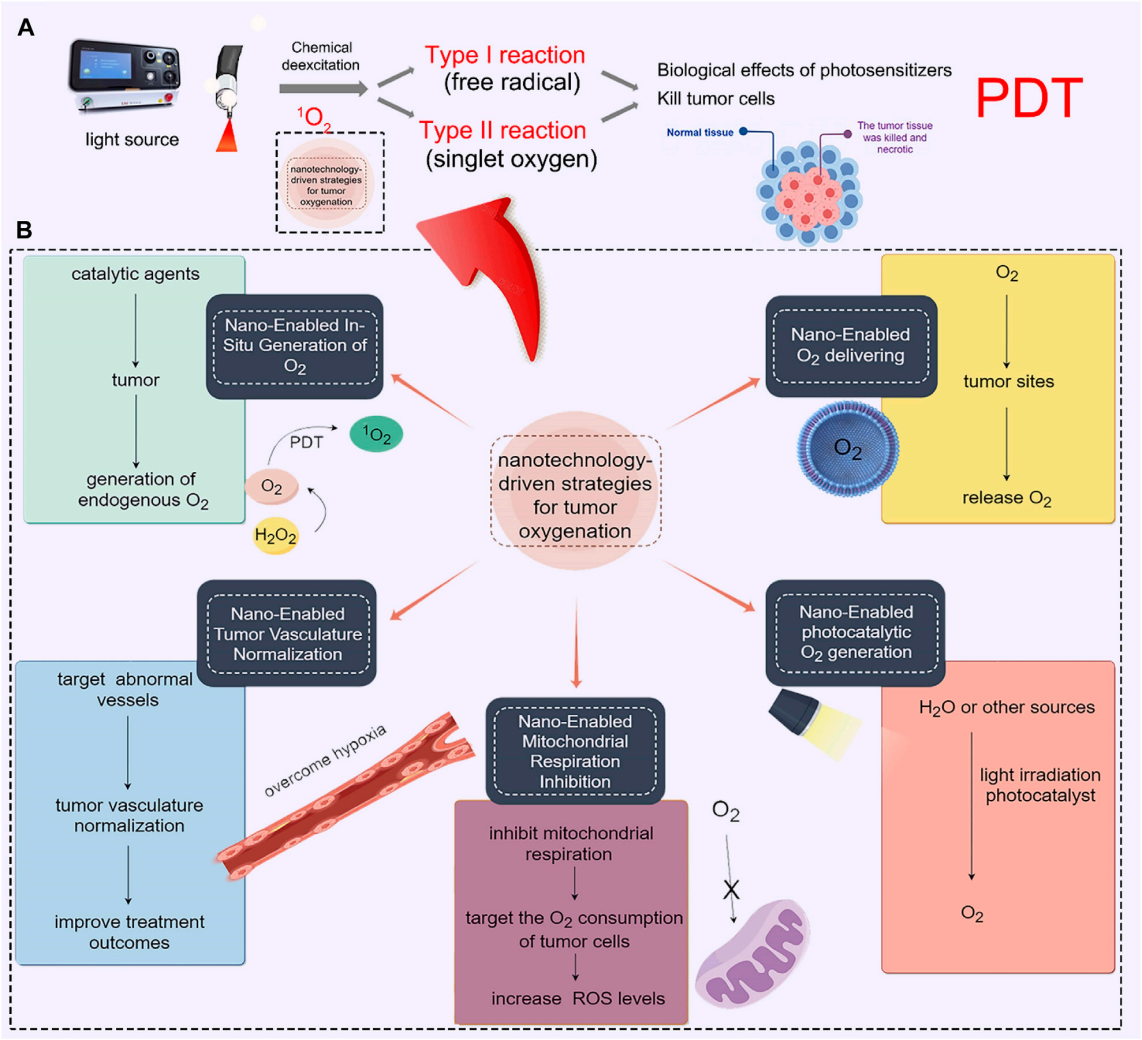
Engineering tumor-oxygenated nanomaterials for enhancing photodynamic therapy (PDT) efficacy. **(A)** Principle of PDT. **(B)** Five strategies for tumor oxygenation, including in-situ O_2_ generation, targeted O_2_ delivery, tumor vasculature normalization, modulation of mitochondrial respiration, and photocatalytic O_2_ generation.

In this comprehensive review, we endeavor to encapsulate the latest developments in nanotechnology-driven strategies for tumor oxygenation (Scheme 1). These strategies encompass in-situ O_2_ production, targeted O_2_ delivery, tumor vasculature modulation, modulation of mitochondrial respiration, and photocatalytic generation of O_2_. We will provide an analysis of their features and limitations, shedding light on their potential applications. Furthermore, we will discuss the existing challenges and potential avenues for further improving the prospects of PDT treatment in the clinical setting. By outlining these challenges and exploring possible solutions, we hope to assist researchers in fully comprehending this emerging field and fuel the development of novel cancer treatments. Through this comprehensive review, we aspire to contribute to the scientific community’s understanding of nano-enabled tumor oxygenation strategies. Ultimately, our aim is to foster advancements in cancer treatment, with the goal of improving patient outcomes and quality of life.

## 2 Nano-enabled in-situ generation of O_2_

**FIGURE 1:**
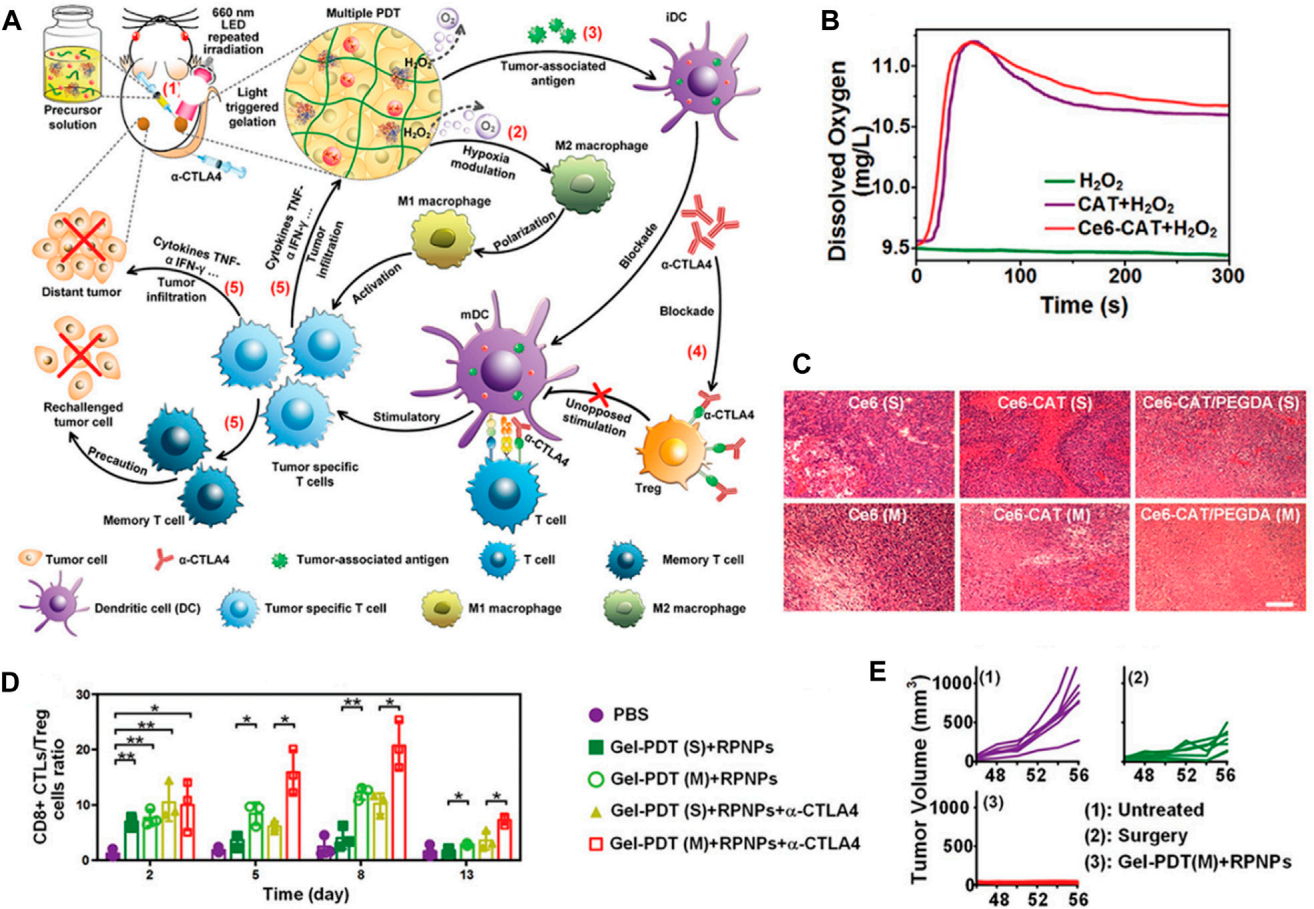
**(A)** The diagram illustrates the mechanism of Gel-based PDT-immunotherapy, which can achieve systemic therapeutic responses by repeated local PDT treatments. **(B)** Shows the production of O_2_ in H_2_O_2_ solutions with either free CAT or Ce6-CAT. **(C)** Displays images of H&E stained tumor slices from different groups of mice on the 7th day. **(D)** Presents the ratios of CD8^+^ CTL to Treg in distant tumors of various groups of mice after each round of PDT (*n* = 3). *, *p* < 0.05; **, *p* < 0.01; ***, *p* < 0.001. **(E)** Shows individual tumor growth curves of rechallenged tumors inoculated 40 days after the elimination of their first tumors by surgery or multiround PDT with RPNPs/Ce6-CAT/PEGDA. ^©^ 2019 WILEY-VCH Verlag GmbH & Co. KGaA, Weinheim.

Tumors often have a hypoxic microenvironment, which hampers the effectiveness of radiation and PDT. Hypoxia, characterized by diminished O_2_ levels, and the elevated presence of hydrogen peroxide (H_2_O_2_) are established hallmarks of the TME (Tunçal et al., 2018; Xiao J. et al., 2023). One such approach involves the in-situ generation of O_2_ within the tumor tissue through catalysis (Huang et al., 2019). By introducing catalytic agents into the tumor, O_2_ can be produced locally, thereby overcoming the O_2_ deficiency. Such as, H_2_O_2_ can efficiently and catalytically decompose into O_2_ and water (H_2_O) (Jiang et al., 2019). This naturally occurring process results in the generation of endogenous O_2_, which significantly enhances the effectiveness of PDT. In spired by this, it is an effective way to precise delivery of catalytic agents to the tumor site, which will greatly increase the possibility of curing the tumor (Nosaka and Nosaka, 2017). In the previous studies, catalytic agents, such as metal nanoparticles or enzymes, were designed to react with endogenous substances present in the TME, such as H_2_O_2_ or glucose (Gao et al., 2021). These reactions generate O_2_ as a byproduct, which can then be utilized to enhance the effectiveness of various treatment modalities. Therefore, extensive efforts have been dedicated to screening and identifying catalysts that can more efficiently decompose H_2_O_2_. These catalysts encompass naturally occurring catalase (CAT) and CAT-like nanoenzymes. As shown in Figure 1, Liu et al. designed a light-triggered *in situ* gelation system containing photosensitizer-modified CAT together with poly (ethylene glycol) double acrylate (PEGDA) as the polymeric matrix (Meng et al., 2019). As the retained CAT triggers the biological decomposition of tumor endogenous H_2_O_2_ to produce O_2_, persistent hypoxia would be relieved, improving PDT efficacy and reversing immunosuppressive TME (Figure 1A). After Ce6-CAT/PEGDA local injection and irradiation, the treatment was found to relieve tumor hypoxia for a longer period of time (Figure 1B). Ce6-CAT/PEGDA exhibited remarkable therapeutic efficacy through a single injection and multiple light treatments, attributed to the long-term hypoxic treatment for improving the efficiency of PDT in destroying tumor cells (Figures 1C–E) (Meng et al., 2019).

**FIGURE 2:**
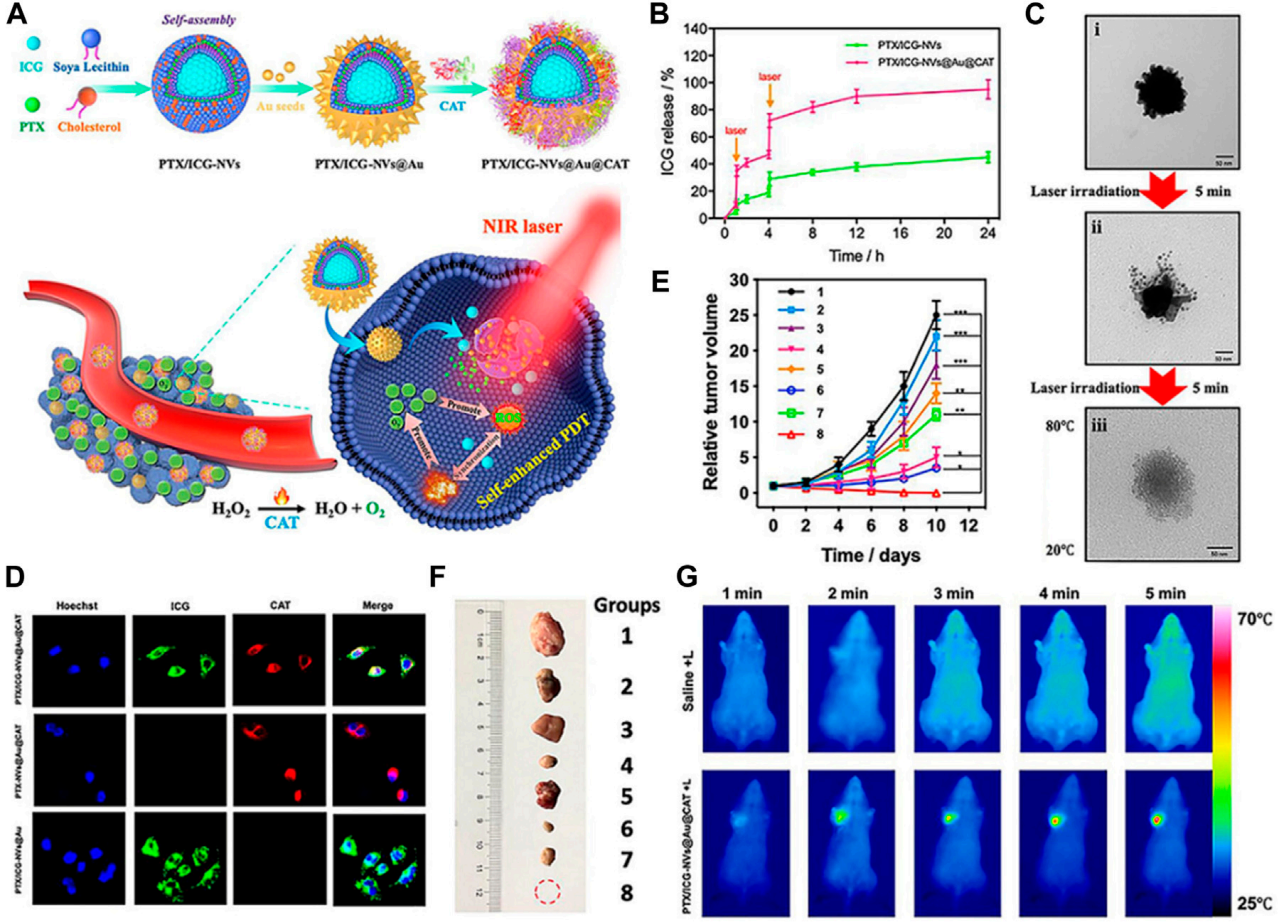
**(A)** Schematic representation of the synthetic process of PTX/ICG-NVs@Au@CAT nanoparticles and their use as a nanomedicine for overcoming hypoxia and achieving self-enhanced PDT combined with chemo, photothermal, and immunotherapy in TME. **(B)** Cumulative release of ICG from PTX/ICG-NVs@Au@CAT nanoparticles under laser irradiation for 5 min at the indicated time points (Arrows indicate the two points of laser irradiation). **(C)** Transmission electron microscopy (TEM) images showing the collapse of PTX/ICG-NVs@Au@CAT nanoparticles under different conditions: (i) non-irradiation, (ii) irradiation once, and (iii) irradiation twice. **(D)** Fluorescence images of HeLa cells incubated with PTX/ICG-NVs@Au@CAT, PTX-NVs@Au@CAT, and PTX/ICG-NVs@Au. The cell nucleus was stained in blue (Hoechst), CAT stained by F-Actin was displayed in red, and ICG was shown in green. **(E)** Tumor growth profiles in different groups of mice after various treatments. **(F)** Representative photographs of tumors after 10 days of treatments. (*p < 0.05, **p < 0.01, ***p < 0.001 compared with the control group). **(G)** Infrared thermal images in the tumor region of tumor-bearing mice treated with saline and PTX/ICG-NVs@Au@CAT followed by laser irradiation (1.5 W cm^−2^ for 5 min). ^©^ 2019 Elsevier B.V. All rights reserved.

Inspired by the excellent well catalytic effect of CAT, Gao et al. employed simple and environmentally friendly chemistry techniques to construct a self-assembled nanosystem (PTX/ICG-NVs@Au@CAT) that serves as a TME-triggered O_2_ nanogenerator (Figure 2A) (He et al., 2019). The researchers evaluated the *in vitro* ROS production capability of PTX/ICG-NVs@Au@CAT under near-infrared (NIR) laser irradiation. The results indicated that the H_2_O_2_ catalysis ability of PTX/ICG-NVs@Au@CAT was enhanced, leading to increased catalytic activity after 808 nm laser irradiation (Figures 2B, C). This enhancement was attributed to the catalytic effect of CAT, which promoted the generation of ROS. As a result, the PTX/ICG-NVs@Au@CAT showed superior photothermal effect under NIR laser irradiation. The increased O_2_ levels within the tumor tissue have several beneficial effects. Firstly, O_2_ is essential for the proper functioning of radiation therapy, as it enhances the production of ROS that damage cancer cells. Secondly, O_2_ can improve the efficacy of certain chemotherapeutic drugs, as some of them require O_2_ to exert their cytotoxic effects. Lastly, O_2_-rich environments can promote the activation of immune cells, leading to enhanced anti-tumor immune responses (Figure 2D). Subsequent *in vivo* findings demonstrated that the O_2_ nanogenerators (PTX/ICG-NVs@Au@CAT) displayed remarkable efficacy in improving the hypoxic condition of tumors and further augmenting immune responses against tumor cells (Figures 2E–G). The infiltration and polarization of macrophages within tumors, along with the levels of IL-10 and IL-12 in the tumor supernatant, collectively indicated a shift from M2 to M1 polarization of tumor-associated macrophages upon treatment with PTX/ICG-NVs@Au@CAT under laser irradiation. Lin et al. developed a multifunctional cascade bioreactor using hollow mesoporous Cu_2_MoS_4_ (CMS) loaded with glucose oxidase (GOx) for synergistic cancer therapy (Chang et al., 2019). The presence of Cu^+^/Cu^2+^ and Mo^4+^/Mo^6+^ redox couples in CMS enables it to effectively generate hydroxyl radicals (·OH) through a Fenton-like reaction, facilitating cancer cell death. Additionally, CMS depletes the overexpressed glutathione (GSH) in the TME, reducing the antioxidant capability of the tumors through its glutathione peroxidase-like activity. Furthermore, CMS exhibits catalase-like activity, reacting with endogenous H_2_O_2_ to produce O_2_, which activates the loaded GOx. Furthermore, the CMS exhibits a CAT-like behavior, enabling it to interact with endogenous H_2_O_2_ and produce O_2_. This process activates the loaded GOx, facilitating the catalyzed oxidation of glucose and simultaneously regenerating H_2_O_2_ within tumor cells. The regenerated H_2_O_2_ plays a crucial role in promoting Fenton-like reactions, thereby enhancing the efficacy of GOx-catalyzed cancer dynamic therapy (CDT). Jiang et al. also developed a hydrogel for phototherapy, known as prolonged O_2_-generating phototherapy hydrogel (POP-Gel), using the “cold method” and incorporating calcium superoxide (CaO_2_) and CAT (Zhou et al., 2019). This O_2_-generating system demonstrated a remarkable capacity for replenishing O_2_ for up to 5 days, effectively mitigating tumor hypoxia. Moreover, the POP-Gel facilitated PDT and exhibited a significantly enhanced production of singlet O_2_, resulting in a heightened ability to inhibit tumor cell growth and induce apoptosis and/or necrosis, both *in vitro* and *in vivo*.

Nanozymes hold great promise for cancer therapy, especially in addressing tumor hypoxia. Some nanozymes can catalyze the generation of oxygen. For example, manganese dioxide nanozymes can catalyze the decomposition of hydrogen peroxide to produce oxygen. These nanozymes can elevate oxygen levels in tumor tissues, thereby alleviating tumor hypoxia. An O_2_-enhanced immunogenic PDT nanozyme was documented (Liang et al., 2018). The nanozyme consisted of a core-shell gold nanocage coated with manganese dioxide (AuNC@MnO_2_, AM) nanoparticles, which acted as O_2_ producers responsive to the TME and generators of ROS triggered by NIR light. This O_2_-enhanced immunogenic PDT approach was utilized against metastatic triple negative breast cancer (mTNBC). The degradation of MnO_2_ provided a sufficient supply of O_2_, resulting in the production of the highest amount of ROS when combined with laser treatment (AM + laser). This increased the apoptosis of tumor cells and exposure of damage-associated molecular patterns (DAMPs), subsequently promoting the maturation of dendritic cells (DCs) and eliciting an immune response. In order to enhance the effectiveness of immunotherapy, Cui et al. developed a nanoplatform (Mn@CaCO_3_/ICG@siRNA) that incorporates laser-mediated photodynamic immunotherapy with supportive immunotherapy, leveraging the superior catalytic activity of MnO_2_ to target hypoxia extracellular matrix (ECM) (Liu et al., 2019). This nanoplatform effectively addresses the challenges posed by the limited therapeutic outcomes of conventional PDT and tumor immune resistance resulting from tumor environmental characteristics. Consequently, it presents a novel approach for combined PDT-immunotherapy, thereby enabling the successful treatment of malignant tumors. Xing et al. also developed a unique photosensitizer-loaded UCNs nanoconjugate (PUN) by integrating manganese dioxide (MnO_2_) nanosheets and hyaluronic acid (HA) biopolymer to improve NIR light-mediated PDT efficacy through attenuating hypoxia status and synergistically reprogramming Tumor-Associated Macrophages (TAMs) populations (Ai et al., 2018). It demonstrated that the HA-modified PUN could act as a mediator to effectively reprogram M2-like TAMs to M1-like macrophages, to prevent tumor relapse after PDT treatment, after overcoming hypoxia by MnO_2_. Furthermore, among various types of nanozymes, iron-based nanozymes have been extensively studied due to their good catalytic activity, low cost, and high stability, and iron is also an essential element for the human body. The iron-based nanozymes (Fe_5_HO_8_·4H_2_O) have the potential to catalyze the Fenton reaction, promoting the conversion of H_2_O_2_ to O_2_ (Fu et al., 2023).

Overall, the use of catalysis to generate O_2_ within tumor tissues holds great potential in improving the effectiveness of cancer treatment (Zetrini et al., 2023). By addressing the hypoxic microenvironment, this approach can enhance the outcomes of radiation therapy, chemotherapy, and immunotherapy, ultimately leading to better patient outcomes (Chen Z. et al., 2023). Further research and development in this field are necessary to optimize the catalytic agents and their delivery methods, but the future looks promising for this innovative approach to cancer treatment (Petrova et al., 2018).

## 3 Nano-enabled O_2_ delivering

Despite the encouraging preclinical and clinical phase I and II findings, the translation of hypoxia-activated prodrugs into clinical practice has thus far encountered limited success (Wang Z. et al., 2021; Yang et al., 2022). Another promising approach involves the targeted delivery of O_2_ to tumor sites, which can enhance the effectiveness of existing therapies (Jiang et al., 2022). The first strategy that scientists thought of is the use of hyperbaric O_2_ therapy (HBOT) (Gottfried et al., 2021). HBOT involves breathing pure O_2_ in a pressurized chamber, which allows for higher O_2_ levels to be dissolved in the bloodstream. This increased O_2_ concentration can then be delivered to tumor tissues, helping overcome tumor hypoxia (Ortega et al., 2021). HBOT has shown promising results in preclinical studies and is currently being evaluated in clinical trials for its potential to enhance cancer treatment outcomes (Zilberman-Itskovich et al., 2022). However, there are potential risks and side effects associated with the HBOT therapy, such as ear and sinus barotrauma, O_2_ toxicity, and claustrophobia. Then, the nanoparticles are designed to encapsulate O_2_ molecules and deliver them directly to tumor tissues. By releasing O_2_ in the TME, these nanoparticles can help increase O_2_ levels and improve the effectiveness of cancer treatments (Thom, 2011; Memar et al., 2019). Additionally, they can enhance the delivery of chemotherapy drugs, as O_2_ is known to enhance the sensitivity of tumor cells to these drugs.

**FIGURE 3:**
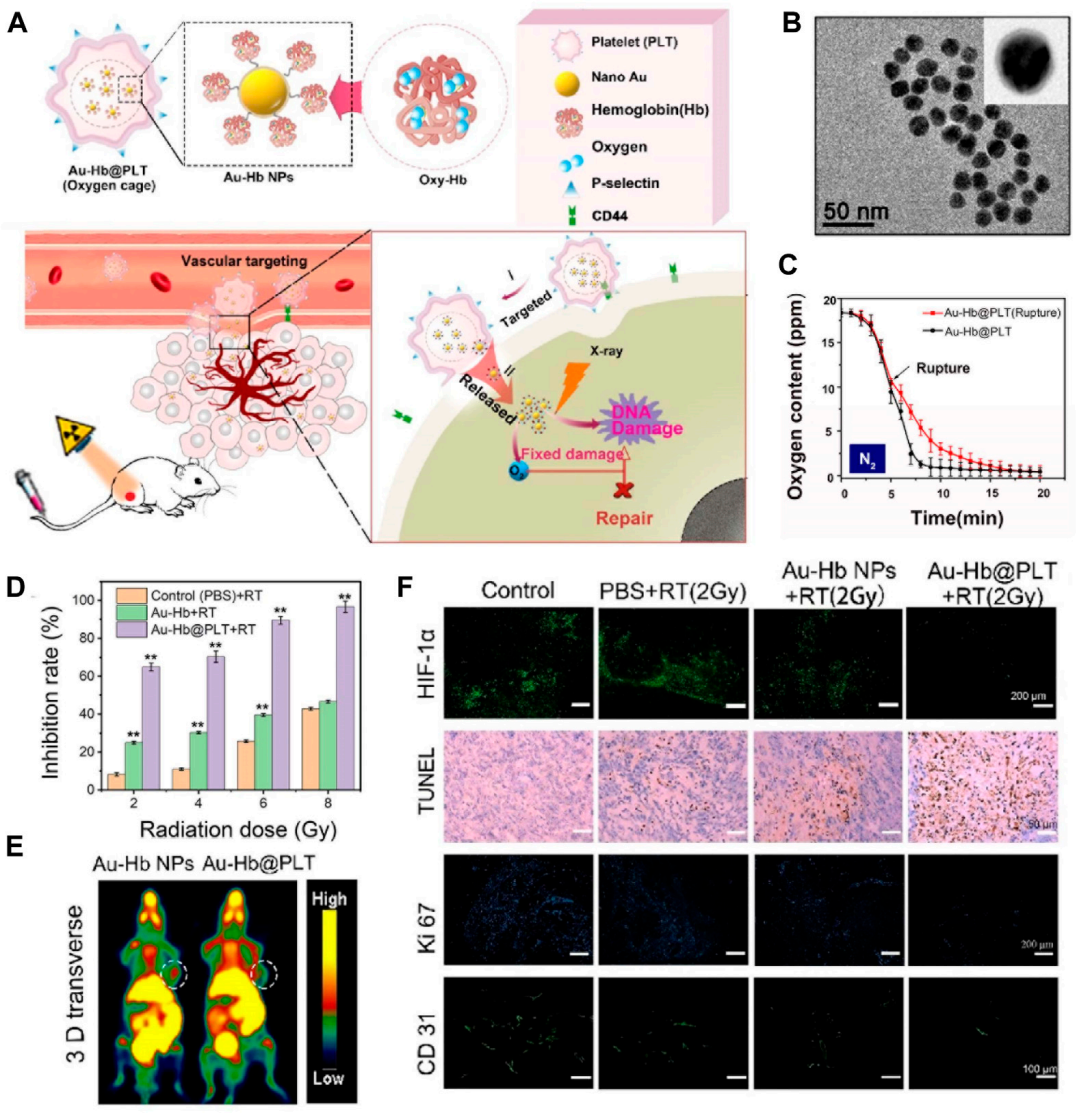
**(A)** Au-Hb@PLT nanoparticles illustrates the concept of Au-Hb@PLT overcoming hypoxia and enhancing the anti-tumor effect in radiation therapy (RT). **(B)** The transmission electron microscopic image of Au-Hb nanoparticles (NPs). **(C)** Au-Hb@PLT nanoparticles presents the measurement of oxygen content in the medium before and after the rupture of Au-Hb@PLT in a nitrogen atmosphere. **(D)** The cytotoxicity of Au-Hb@PLT under X-ray irradiation, with statistical significance indicated by **p < 0.01 compared to the control + RT group. **(E)** Positron emission tomography (PET) images of mice treated with Au-Hb NPs or Au-Hb@PLT. **(F)** Micrographs of HIF-1α staining, TUNEL, Ki-67, and CD31 from different groups at day 28 after treatment. ^©^ 2020 American Chemical Society.

Carrier nutrients, such as nanoparticles or liposomes, can be utilized to transport O_2_ to tumor sites (Zhang et al., 2018; Li et al., 2019). These carriers have the ability to encapsulate and protect O_2_ molecules, ensuring their safe delivery to the TME (Song et al., 2017; Xia et al., 2023). Additionally, carrier nutrients can be engineered to specifically target tumor cells, further enhancing their therapeutic potential. Hu et al. developed a novel strategy that combines the mitigation of hypoxia in tumor tissue through O_2_ delivery and the enhancement of radiotherapy through the use of radiosensitizers, as shown in Figure 3 (Xia et al., 2020). This was achieved by encapsulating an O_2_ carrier (hemoglobin, Hb) along with nano-Au radiosensitizers within platelets through intermolecular disulfide coupling, resulting in the formation of platelet-coated particles (Au-Hb@PLT) (Figure 3A). The presence of P-selectin on the platelet surface facilitated specific binding to cancer cells, as these cells exhibited an upregulation of CD44 on their surface. These Au-Hb@PLT nanoparticles serve as O_2_ reservoirs and have the ability to directly transport molecular O_2_ and radiosensitizers to the tumor tissue, effectively addressing the issue of hypoxia in the tumor cells. Furthermore, the reduced size of Au-Hb@PLT enables them to be activated by the tumor cells and penetrate deeply into the tumor tissue (Figures 3B, C). The results from immunofluorescence analysis of tumor slices and PET images of mice treated with Au-Hb NPs or Au-Hb@PLT clearly demonstrate the significant enhancement of the antitumor effect in radiation therapy at a dosage of 2 Gy X-ray (Figures 3D–F). From those results, the hypoxia could be relieved by the molecular O_2_ from Hb and resulted good RT outcomes.

**FIGURE 4:**
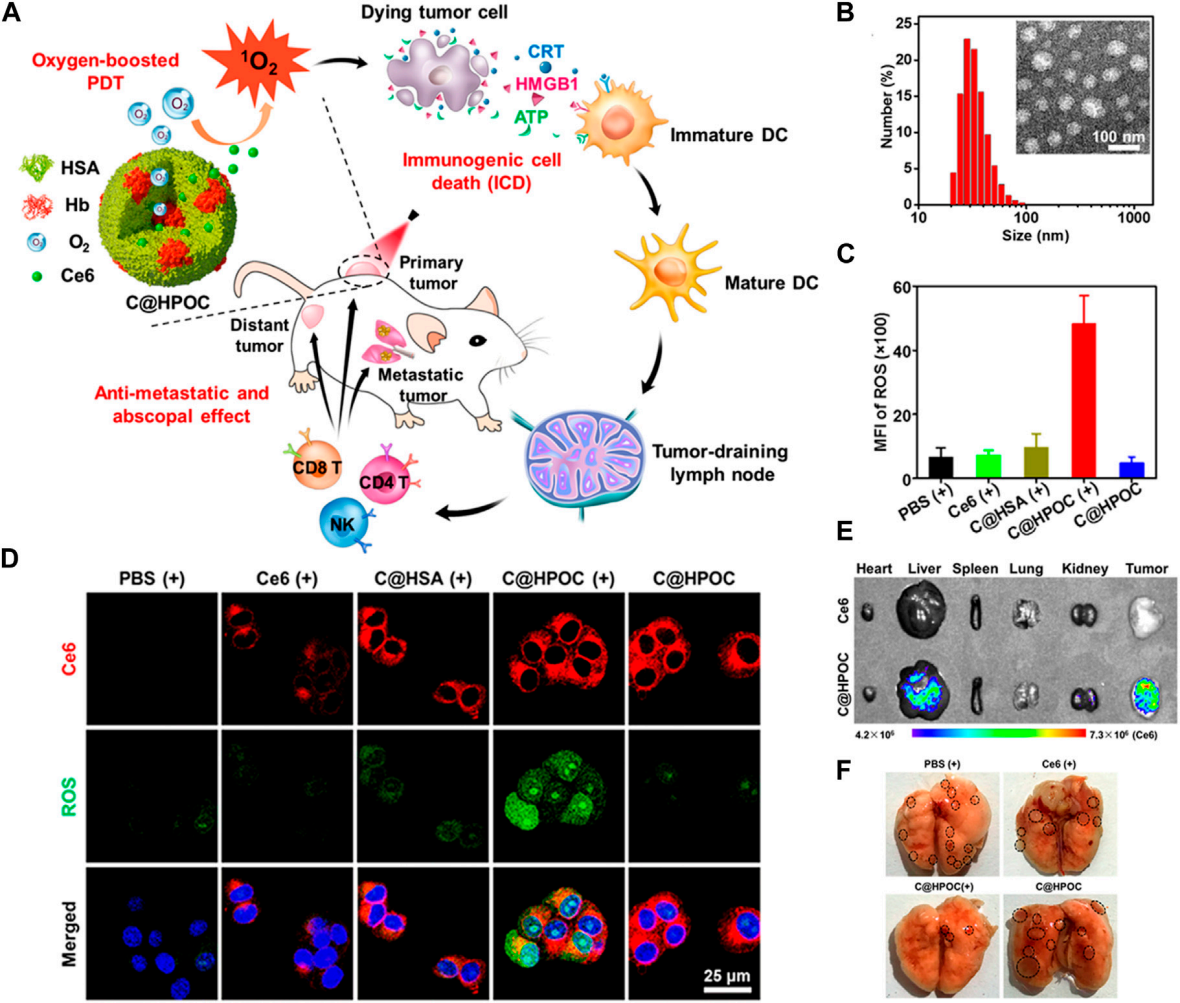
**(A)** Schematic representation of O_2_-enhanced immunogenic PDT using C@HPOC to induce anti-metastatic and abscopal effects. **(B)** Size distribution of C@HPOC measured using dynamic light scattering (DLS). Inset: TEM image of C@HPOC. **(C)** Quantification of reactive oxygen species (ROS) production in 4T1 tumor cells using flow cytometry after different treatments. **(D)** Confocal images showing cellular uptake and ROS generation in 4T1 tumor cells. **(E)** Fluorescence images of major organs and tumors 24 h after intravenous injection of free Ce6 or C@HPOC (*n* = 3). **(F)** Photographs of metastatic nodules in the lungs. The black circles indicate the metastatic nodules. ^©^ 2018 American Chemical Society.

Once the carrier nutrients reach the tumor site, they release O_2_ through various mechanisms. One approach involves the use of enzymatic reactions tri ggered by the TME, which can break down the carrier nutrients and release O_2_. Another method involves the use of external stimuli, such as light or heat, to trigger the release of O_2_ from the carriers (Li Y. et al., 2020). These mechanisms ensure that O_2_ is delivered precisely to the tumor cells, maximizing its therapeutic impact. Cai et al. hybridized human serum albumin with hemoglobin through intermolecular disulfide bonds to develop a Ce6 coated mixed protein- O_2_ nano-capsules (C@HPOC) for O_2_ self-contained PDT, which realized tumor targeted co-delivery of photosensitizer and O_2_, and significantly alleviated tumor hypoxia (Figures 4A, B) (Chen et al., 2018). Under laser irradiation, O_2_ self-supplied nanoparticles (C@HPOC) elevated the generation of cytotoxic ^1^O2 and moreover triggered immunogenic cell death (ICD) (Figure 4C). The ROS production ability of C@HPOC in 4T1 tumor cells was studied by photoacoustic imaging, confocal laser scanning microscopy (CLSM) and flow cytometry. Immunofluorescence staining and flow cytometry were used to detect the modulating immunosuppressive TME of C@HPOC on tumor oxygenation and promote the infiltration of CD8^+^ T cells in the tumor (Figures 4D–F). This study might present an inspiration for improving the therapeutic outcomes and prognosis of metastatic cancers in clinics (Chen et al., 2018).

**FIGURE 5:**
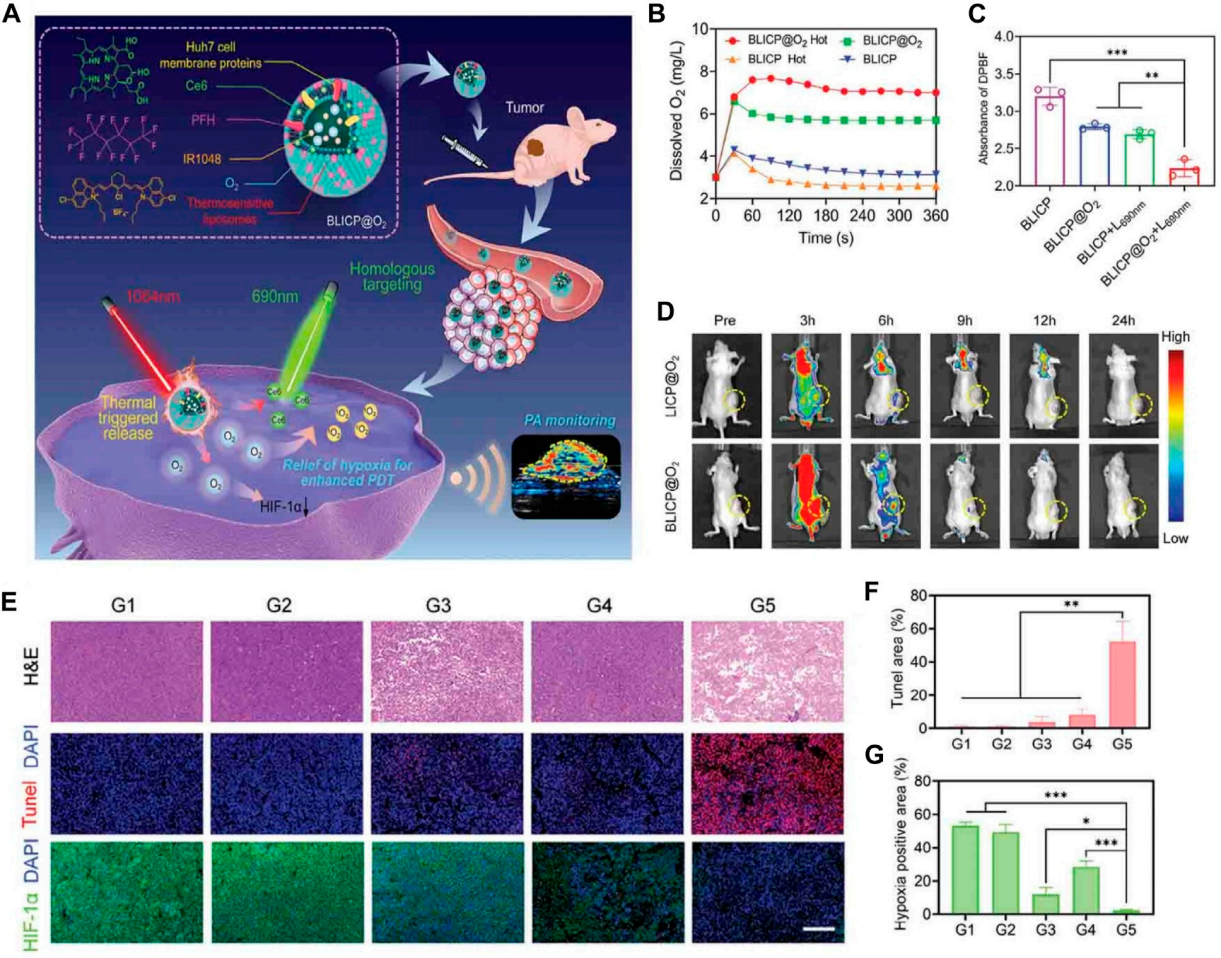
**(A)** Schematic representation of the NIR-II photoacoustic guided-controllable oxygen (O_2_) supply of biomimetic nanoparticles for highly specific hepatocellular carcinoma (HCC) photodynamic therapy (PDT). **(B)** Changes in O_2_ concentration in BLICP@O_2_ and BLICP solutions under different treatments. **(C)** Generation of reactive oxygen species (ROS) by BLICP@O_2_ and BLICP solution with or without exposure to a 690 nm laser (1 W cm^−2^). ROS generation was determined by measuring the attenuation of DPBF absorbance at 450 nm. **(D)** Real-time fluorescence images of the entire body (CLICP@O_2_ = CBLICP@O_2_ = CCe_6_ = 0.5 mg mL^−1^). Tumor regions are indicated by yellow circles. **(E)** Hematoxylin and eosin, (H&E) staining, terminal deoxynucleotidyl transferase dUTP nick end labeling (TUNEL) staining, and hypoxia-inducible factor-1 alpha immunofluorescence of tumor slices collected from different groups after various treatments. **(F)** Quantification of TUNEL-positive cells in tumors after various treatments. **(G)** Quantification of HIF-1α expression in tumors after various treatments (*p < 0.05, ***p < 0.001). ^©^ 2023 The Author(s). Published by Elsevier B.V.

Fang et al. have developed a biomimetic O_2_ delivery system known as BLICP@O_2_, which aims to deliver O_2_ specifically to tumors and control its release, as shown in Figure 5A (Zeng et al., 2023). This system employs a combination of hybrid tumor cell membranes and thermosensitive liposomes as carriers for O_2_, incorporating the NIR-II dye IR1048, the photosensitizer chlorin e6 (Ce6), and perfluorohexane. The release of O_2_ was evaluated using contrast-enhanced ultrasound (CEUS) imaging, revealing that BLICP@O_2_ successfully generated bubbles containing O_2_ when thermally triggered (Figure 5B). A 3D liver cancer tumor spheroid model was constructed using Huh7 cells to simulate the hypoxic environment of solid tumors. It was found that after 1064 nm laser irradiation tumor spherical hypoxia was effectively alleviated treated with BLICP@O_2_ (Figure 5C). Moreover, BLICP@O_2_ demonstrated a remarkable capacity to convert light energy into thermal energy, consequently stimulating the release of O_2_ via the photothermal effect. This characteristic can be harnessed to enhance the effectiveness of PDT. The delivery of O_2_ to tumor sites can significantly improve the efficacy of existing cancer treatments (Figures 5D–G). O_2_-rich environments enhance the sensitivity of tumor cells to radiation therapy, making them more susceptible to radiation-induced cell death. Additionally, O_2_ can enhance the effectiveness of chemotherapy drugs by increasing their cytotoxicity towards tumor cells. Therefore, the combination of O_2_ delivery and conventional cancer treatments can lead to improved tumor regression and patient outcomes.

The targeted delivery of O_2_ to tumor sites through carrier nutrients represents a promising strategy to enhance tumor treatment efficacy. By increasing O_2_ levels in the TME, it can improve the sensitivity of tumor cells to radiation therapy and chemotherapy. Further research and development in this field hold great potential for revolutionizing cancer treatment and improving patient survival rates (Sharma et al., 2021). Despite promising preclinical results, targeted delivery of O_2_ to tumor sites has had limited success in clinical trials. This is likely due to the challenges, such as poor tumor penetration, toxicity to healthy tissues, as well as the difficulty in manufacturing and administering these systems (Xiao Y. et al., 2023).

## 4 Nano-enabled tumor vasculature normalization

Tumor vasculature normalization is a promising strategy to enhance the efficacy of tumor treatment (Magnussen and Mills, 2021; Yang et al., 2021). In the TME, abnormal blood vessels often develop, leading to poor blood flow and limited drug delivery to the tumor site. However, by targeting these abnormal vessels and restoring their functionality, tumor vasculature normalization can improve the delivery of therapeutic agents to the tumor (Choi and Jung, 2023).

**FIGURE 6:**
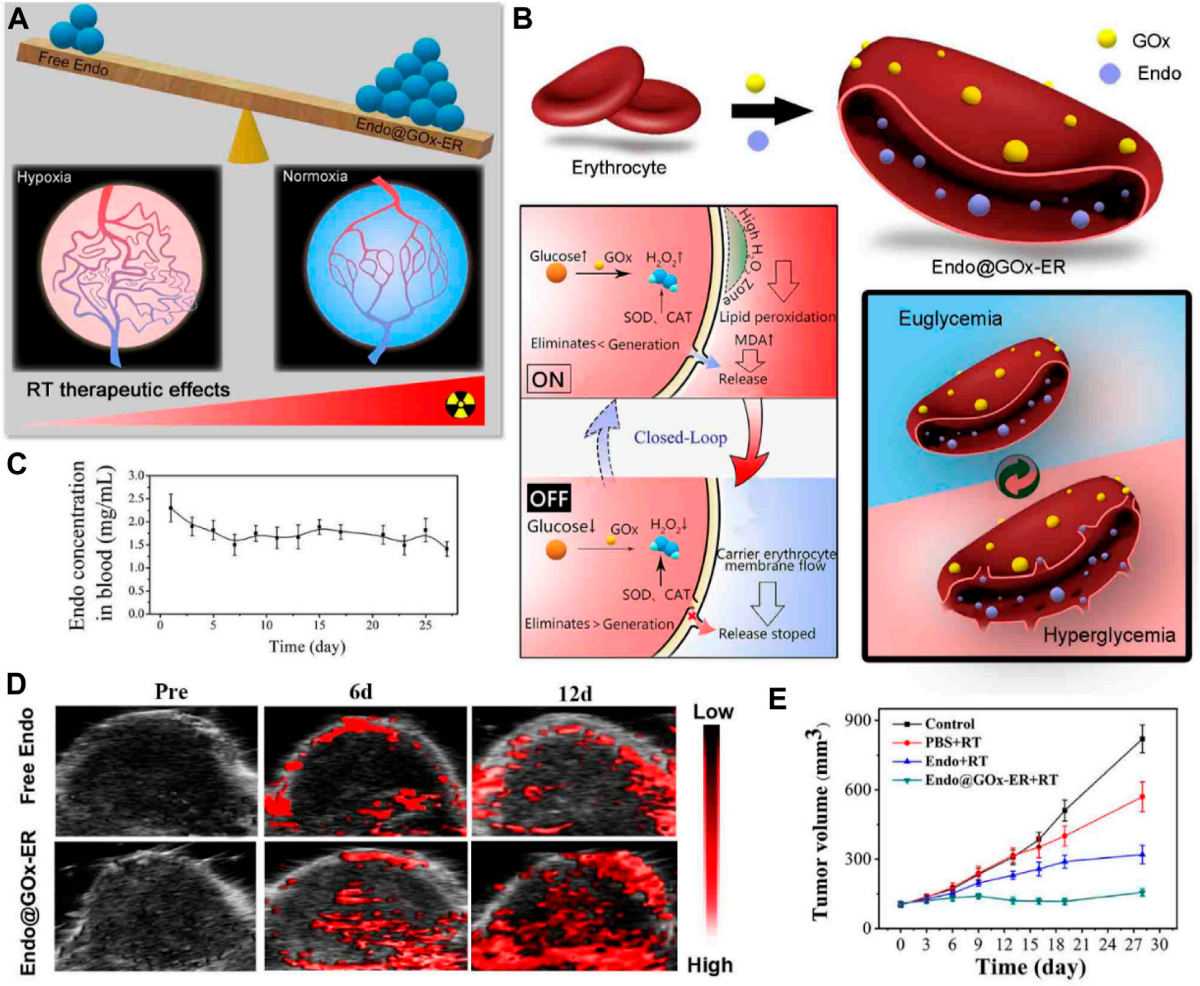
**(A)** Illustration demonstrating a platform inspired by erythrocytes and activated by glucose to overcome hypoxia-restrained radiotherapy. **(B)** Creation of erythrocytes conjugated with glucose oxidase (GOx) and loaded with endostatin (Endo@GOx-ER). The diagram depicts the controlled release of Endo from Endo@GOx-ER in response to glucose levels. **(C)** Graph showing the concentration of Endo@GOx-ER in the plasma over time. **(D)** Photoacoustic imaging of the tumor region. The signal intensity of oxyhemoglobin increased in the Endo@GOx-ER group, indicating that the stable levels of Endo in this group effectively improved tumor oxygenation. **(E)** Comparison of relative tumor growth curves in mice after intravenous administration of different treatments over a period of 28 days. ^©^ 2020 American Chemical Society.

One approach to achieving tumor vasculature normalization is through the use of anti-angiogenic agents (Schaaf et al., 2018). These drugs inhibit the formation of new blood vessels and can also help normalize the existing vasculature. By reducing vessel leakiness and increasing blood flow, anti-angiogenic agents enhance the delivery of chemotherapy drugs and immune cells to the tumor, thereby improving treatment outcomes. Endostar, a molecular targeted anti-tumor drug that has been autonomously developed in China, is extensively employed in the therapeutic management of lung cancer and various other malignancies (Xu et al., 2018; Ma et al., 2021). To achieve a stable effective blood concentration, it is recommended that Endostar is administered once per day over 4 hours. To reduce the need for repeated drug administration and overcome off-target effects, Xia et al. reported a glucose-regulated drug release platform, Endostar-loaded GOx-conjugated erythrocytes (Endo@GOx-ER) (Figure 6A) (Huang et al., 2020). During euglycemia, Endo was stored in the ER drug delivery system. In the context of hyperglycemia, the presence of H_2_O_2_ resulting from the catalytic oxidation of glucose by GOx would facilitate the development of pores on the membranes of red blood cells (Fu et al., 2022). Conversely, in normoglycemia, the release of Endo may be inhibited due to the closure of these pores (Figure 6B). The repeated cycles resulted in sustained high Endostar plasma levels, which dramatically normalized the tumor vasculature and chronically reversed tumor hypoxia (Figure 6C). The *in vivo* results implied the Endo@GOx-ER induced vascular normalization effectively conquered the tumor hypoxia, which improves therapeutic outcomes when combined with radiotherapy (Figures 6D, E).

**FIGURE 7:**
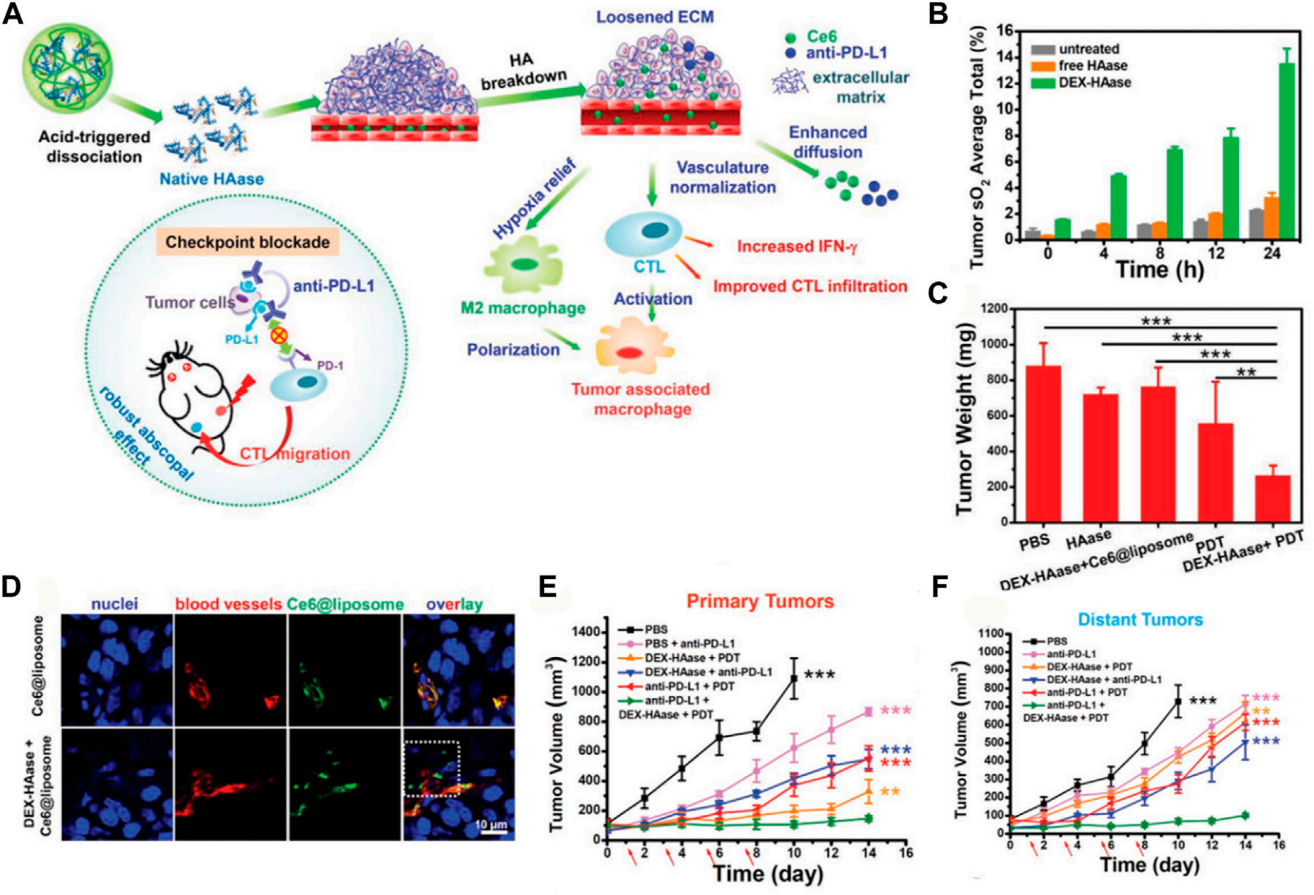
**(A)** The proposed mechanism for enhancing photodynamic therapy (PDT) and anti-tumor immune responses using the DEX-HAase adjuvant and PD-L1 checkpoint blockade. **(B)** Measurement of tumor oxyhemoglobin saturation over time in different groups. **(C)** Average tumor weights recorded at day 14 after various treatments. **(D)** Confocal fluorescence images of tumor slices showing the fluorescence of Ce6, DAPI stained nuclei, and anti-CD31 stained blood vessels. **(E)** Growth curves of primary tumors in different groups after various treatments. **(F)** Growth curves of distant tumors in different groups after various treatments. The arrows indicate the time points of anti-PD-L1 administration on days 1, 3, 5, and 7. ^©^ 2019 WILEY-VCH Verlag GmbH & Co. KGaA, Weinheim.

Hyaluronan is highly expressed in most solid tumors, which facilitates tumor hypoxia. An enhancing penetration of O_2_ therapeutic strategy was proposed by Liu et al. (Wang et al., 2019). Hyaluronidase (HAase, EC 3.2.1.35), an enzyme which cleaves the polysaccharide HA in the ECM of connective tissue, was used to loosen the ECM structure. A study was conducted to enhance the effectiveness of treatment by implementing a pH-responsive HAase delivery and release system. This system aimed to loosen the tumor ECM, regulate the TME, and improve the therapeutic outcomes of PDT and combined photodynamic-immunotherapy (Figure 7A). To achieve this, the researchers utilized dextran (DEX), a commonly used biocompatible polymer, as a protective agent to conjugate with HAase using a pH-sensitive traceless linker known as 3-(bromomethyl)-4-methyl-2,5-furandione. Subsequently, DEX-HAase nanoparticles exhibit inhibited enzyme activity at physiological pH levels, but can release unaltered free HAase at weakly acidic pH levels within the tumor. This subsequently induces the degradation of HA, resulting in the loosening of the ECM structure and facilitating improved penetration of O_2_ and therapeutic agents (Figure 7B). The findings from confocal fluorescence images of tumor slices and tumor growth curves provide evidence that DEX-HAase treatment effectively alleviates tumor hypoxia (Figures 7C–F). The significant reduction in tumor hypoxia would enhance the efficacy of nanoparticle-based PDT, concurrently reversing the immunosuppressive TME to augment cancer immunotherapy (Wang et al., 2019).

Another strategy for tumor vasculature normalization involves the use of physical interventions, such as radiation therapy or focused ultrasound (Chen K. T. et al., 2023; Leong et al., 2023). These techniques can selectively damage the abnormal blood vessels, leading to their regression and subsequent normalization. This normalization process improves blood flow and oxygenation within the tumor, making it more susceptible to chemotherapy and radiation therapy (Hughes et al., 2019; Belcher et al., 2020). Tumor vasculature normalization holds great potential for improving the efficacy of tumor treatment. By restoring the functionality of abnormal blood vessels, it enhances drug delivery, oxygenation, and immune cell infiltration within the TME. This approach can significantly enhance the effectiveness of chemotherapy, radiation therapy, and immunotherapy, ultimately leading to better outcomes for cancer patients (Sharma et al., 2023).

## 5 Nano-enabled mitochondrial respiration inhibition

Researchers have been exploring more effective strategies to enhance the effectiveness of cancer treatment (Yang et al., 2020). One promising approach involves targeting the O_2_ consumption of tumor cells (Zhao et al., 2022; Ganji et al., 2023). Tumor cells have a high metabolic rate and rely heavily on O_2_ to sustain their rapid growth. By inhibiting mitochondrial respiration, scientists aim to disrupt the energy supply to cancer cells, ultimately leading to their demise (Yang et al., 2018; Sun et al., 2022). Mitochondrial respiration is a crucial process that enables cells to generate energy in the form of adenosine triphosphate (ATP). Cancer cells heavily rely on this energy production pathway to sustain their rapid growth and proliferation (Wang Q. et al., 2023; Zhao et al., 2023). Therefore, by selectively inhibiting mitochondrial respiration, it is possible to selectively target cancer cells while sparing healthy cells.

**FIGURE 8:**
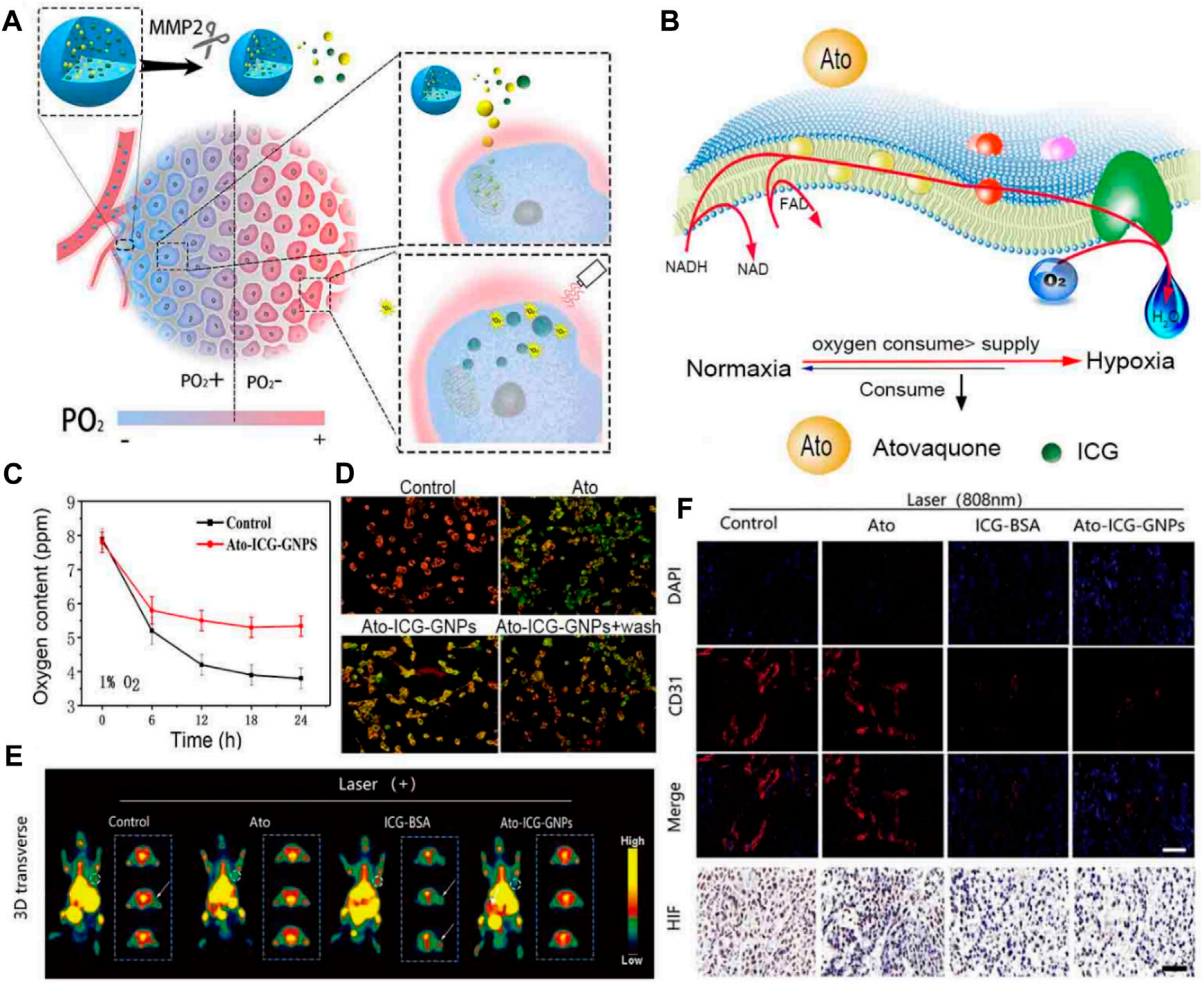
**(A)** An illustration demonstrating how the Ato-ICG-GNPs can enhance the effectiveness of PDT treatment in killing tumors. **(B)** A diagram showing the wide-ranging impact of Ato on OXPHOS, and the mechanism by which Ato-ICG-GNPs assist in reversing hypoxia. **(C)** Changes in the oxygen content. **(D)** The results of the mitochondrial membrane potential in HeLa cells treated with PBS, Ato, Ato-ICG-GNPs (in the presence of 0.2 × 10^−6^ M MMP-2) for 6 h. Subsequently, the cells treated with Ato-ICG-GNPs were washed with PBS to determine if the effects of Ato can be reversed (Ato-ICG-GNPs + wash). Scale bar: 10 µm *p < 0.05, **p < 0.01. **(E)** PET images of mice receiving different treatments. **(F)** Representative immunofluorescence images of tumor tissue sections stained with the nucleus (blue) and blood vessel marker, CD31 (red). ^©^ 2019 Published by Elsevier Ltd.

The hypoxia observed in tumor cells is primarily attributed to the excessive O_2_ consumption that occurs following the generation of ATP during mitochondrial-associated oxidative phosphorylation (Grün et al., 2022). Several studies have shown promising results in preclinical models, where inhibiting mitochondrial respiration has led to increased sensitivity of cancer cells to conventional therapies such as chemotherapy and radiation (Ren et al., 2022). This approach not only enhances the effectiveness of these treatments but also reduces the likelihood of resistance development. One of the magical effects of metformin (Met) is the inhibition of mitochondrial respiration, which is also used in tumor treatment. In order to improve the targeting property, Teng et al. developed platelet membranes (PM) as nano-carriers to co-encapsulate Met and IR780 (PM-IR780-Met NPs) (Figures 8A, B) (Mai et al., 2020). As a PDT agent, IR780 could effectively kill the tumor by producing toxic ROS, while the introduction of Met inhibited mitochondrial respiration and reduced tumor O_2_ consumption, thereby evoking an O_2_-boosted PDT and propelling the immunogenic cell death (ICD)-based immunogenic pathway. The *in vitro* and *in vivo* results strongly demonstrated that PM-IR780-Met NPs could inhibit the cell respiration by the detection of the oxygen consumption rate (OCR) of 4T1 cells. Then, confocal laser scanning microscopy and PET images were used to qualitatively examine ROS generation in 4T1 cells illustrating that the addition of Met could boost the PDT outcome (Figures 8C–F) (Mai et al., 2020).

**FIGURE 9:**
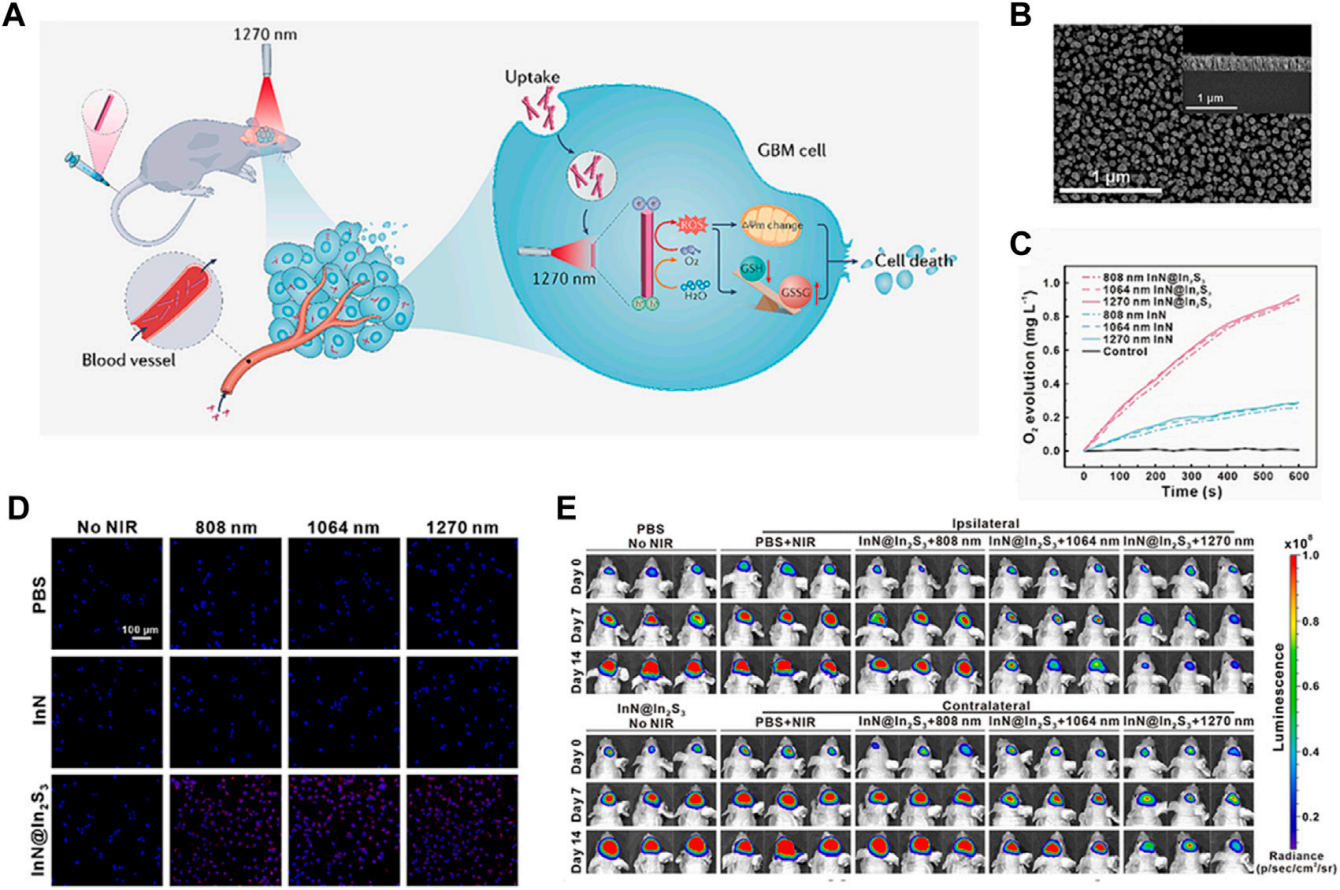
**(A)** When InN@In_2_S_3_ penetrates through the BBB and accumulates in GBM cells, it can generate a large amount of O_2_ and ROS when exposed to 1,270 nm laser irradiation. This leads to disruption of the mitochondrial membrane potential and redox reaction, ultimately causing cell death. **(B)** The SEM images show the morphology of InN@In2S3, with corresponding high-resolution TEM images shown in the insets. **(C)** The treatments result in the production of O_2_. **(D)** The cellular O_2_ levels of U87MG cells were measured using [Ru (dpp)_3_] Cl_2_ staining. **(E)** The tumor growth before and after phototherapy was monitored using bioluminescence imaging. Each group consisted of 6 samples. ^©^ 2023 The Author(s). Published by Elsevier B.V.

Atovaquone (Ato) is a drug that is known to interfere with the cellular respiration mechanism (Ashton et al., 2016). Cellular respiration is the process by which cells generate energy in the form of ATP through the breakdown of glucose and other molecules. Atovaquone specifically targets the mitochondria, which are the powerhouses of the cell responsible for producing ATP (Wang S. et al., 2021). Hu et al. devised a multistage delivery system, termed Ato-ICG-GNPs, capable of reversing hypoxia. This system encapsulates Ato and indocyanine green-bovine serum albumin (ICG-BSA) nanocomplex via a size-shrinkable gelatin carrier (Figure 9A) (Xia et al., 2019). Upon entering the TME, these particles underwent rapid degradation facilitated by the enzyme matrix metallopeptidase 2 (MMP-2), whose expression is abnormally elevated in tumors. This process resulted in the release of ICG-BSA and Ato molecules, which were then directed towards O_2_-deprived regions (Figures 9B, C). Evaluating the mitochondrial membrane potential of HeLa cells using the cyanine dye JC-1 revealed a depolarized mitochondrial membrane in cells that received Ato. The *in vitro* and *in vivo* results showed that the released Ato inhibits the activity of OXPHOS to suppress the consumption of O_2_, remodeling the niche toward one best fitting the action of PDT mediated by ICG (Figures 9D–E). There is optimism that the implementation of this strategy may provide valuable insights into the advancement of PDT-adjuvant therapy for future generations (Xia et al., 2019). Radiation therapy relies on the production of ROS to induce DNA damage in tumor cells. Several studies have shown that reducing the O_2_ consumption of tumor cells can enhance the efficacy of various cancer therapies. By suppressing the O_2_ consumption of tumor cells, it can create a more favorable environment for radiation therapy, allowing the ROS to accumulate and maximize their cytotoxic effects (Shen et al., 2021). Furthermore, inhibiting O_2_ consumption can also enhance the effectiveness of chemotherapy. Many chemotherapeutic drugs rely on the generation of ROS to induce cell death. However, tumor cells with high O_2_ consumption can quickly neutralize these ROS, rendering the treatment less effective. By targeting the O_2_ consumption of tumor cells, it can increase the intracellular ROS levels and sensitize the tumor cells to chemotherapy, leading to improved treatment outcomes.

Metformin has also demonstrated potential applications in addressing tumor hypoxia, a hallmark feature of the tumor microenvironment that contributes to tumor progression, metastasis, and resistance to therapy. Metformin reprograms tumor metabolism by reducing glucose uptake and glycolysis, the primary energy source for many cancer cells. This metabolic shift towards oxidative phosphorylation can limit tumor growth and survival in hypoxic environments, where glycolysis is often impaired (Han et al., 2022).

Overall, by inhibiting the O_2_ consumption of tumor cells, it can potentially enhance the effectiveness of cancer treatment (Yu et al., 2019; Wang D. et al., 2020). This approach has the potential to improve the outcomes of radiation therapy and chemotherapy, offering new possibilities for combating cancer and improving patient survival rates. While the concept of targeting mitochondrial respiration for cancer treatment is still in its early stages, it holds great potential for improving the efficacy of existing therapies (Yu et al., 2019). Further research and clinical trials are needed to fully understand the mechanisms involved and to develop safe and effective strategies for inhibiting mitochondrial respiration in cancer cells.

## 6 Nano-enabled photocatalytic O_2_ generation

Photocatalytic O_2_ generation refers to the process of using a photocatalyst to generate O_2_ from H_2_O or other sources under light irradiation (Wang L. et al., 2022). This technique has gained significant attention in the field of cancer research as it has the potential to enhance tumor PDT (Wang N. et al., 2023). Photocatalytic O_2_ generation can address this limitation by providing a local source of O_2_ within the tumor (Li W. et al., 2020; Wang X. et al., 2022). By using a photocatalyst, such as a semiconductor material, light energy can be harnessed to split H_2_O molecules and release O_2_ (Hu et al., 2022). This O_2_ can then be utilized by the photosensitizer during PDT, enhancing its efficacy.

**FIGURE 10:**
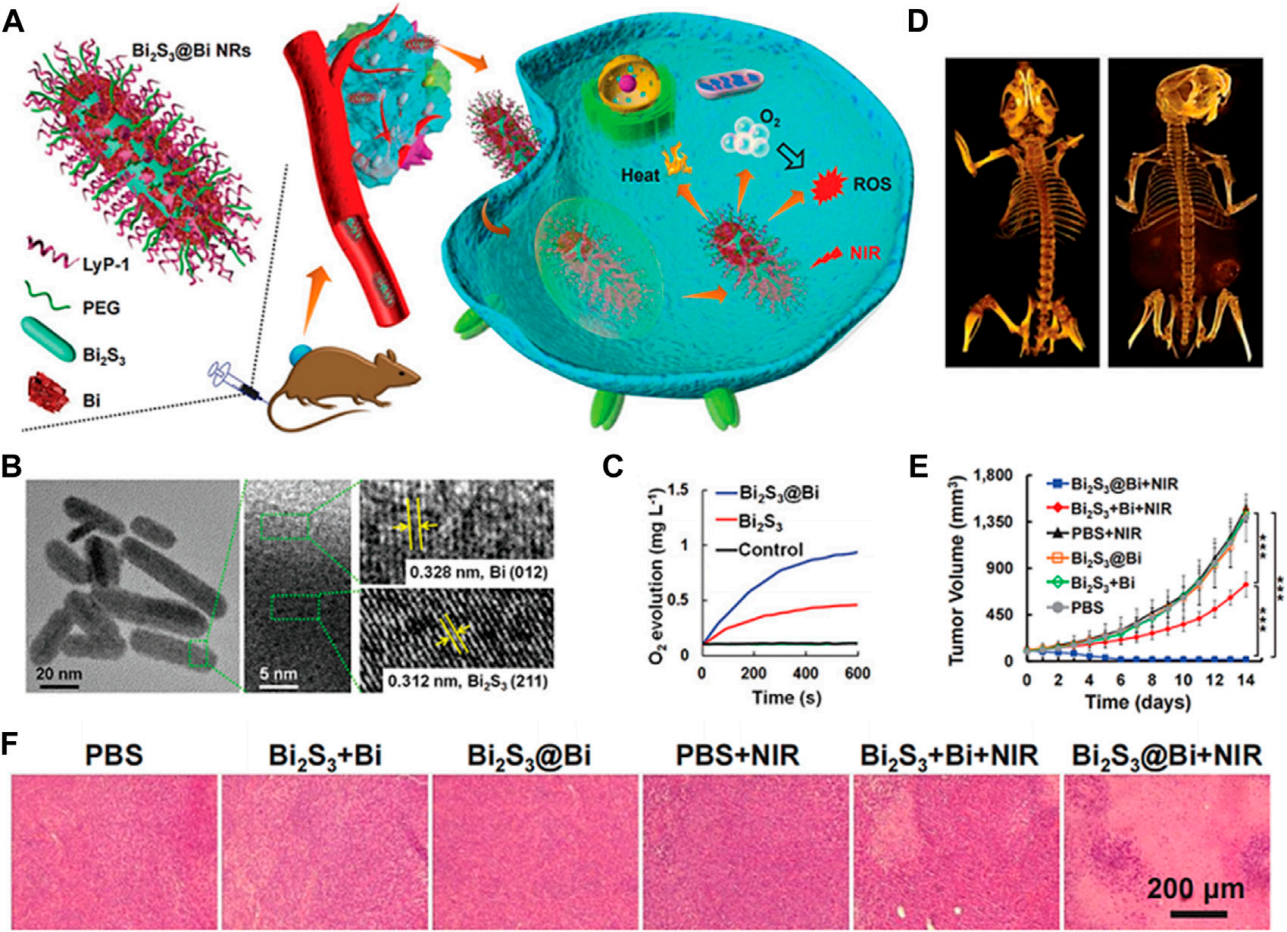
**(A)** Strategy of Bi_2_S_3_@Bi NRs for *in vivo* weight reduction by simultaneously providing O_2_ and generating ROS under NIR laser exposure. **(B)** Transmission electron microscopy (TEM) and high-resolution TEM (HRTEM) images of Bi_2_S_3_@Bi NRs. **(C)** Oxygen (O_2_) generation curves. **(D)** Computed tomography (CT) images of mice before and after intravenous injection with Bi_2_S_3_@Bi NRs (20 mg kg^−1^ mouse) for 12 h. **(E)** Tumor growth curves of mice injected with Bi_2_S_3_ + Bi or Bi_2_S_3_@Bi NRs (20 mg kg^−1^ mouse) with or without 808 nm laser exposure (0.5 W cm^−2^, 5 min); *p < 0.05, ***p < 0.001, analyzed using two-sided Student’s heteroscedastic t-test. **(F)** Hematoxylin and eosin, (H&E) stained tumor sections obtained 14 days after injection. ^©^ 2020 WILEY-VCH Verlag GmbH & Co. KGaA, Weinheim.

Several studies have demonstrated the potential of photocatalytic O_2_ generation in improving tumor PDT (Mu et al., 2023). For example, researchers have developed various photocatalytic systems, such as titanium dioxide (TiO_2_) nanoparticles, that can efficiently generate O_2_ under light irradiation (Çeşmeli and Biray Avci, 2019; Mu et al., 2023). These systems have been shown to enhance the production of ROS and enhance the therapeutic outcome of PDT in cancer cells and animal models. Guo’s group designed a photosensitizer (InN@In_2_S_3_ core-shell nanorod) in order to guarantee an adequate depth of light penetration and O_2_ supply to facilitate PDT clinical treatment of glioblastoma (GBM) (Figure 10A) (Liu et al., 2024). The tailored InN@In_2_S_3_ was synthesized using molecular beam epitaxy (MBE) and annealing methods, which showed more effective separation of electron holes owing to its II heterojunction (Figure 10B). The separated holes catalyze the generation of O_2_ from H_2_O, ensuring an adequate supply of O_2_ (Figure 10C). The ESR analysis using DMSO as the probe revealed the presence of typical signals associated with O_2_·^-^ and OH·, indicating that InN@In_2_S_3_ has the potential to be an effective photosensitizer for tumor therapy. Furthermore, the cellular evaluation demonstrated the ability of InN@In_2_S_3_ to generate O_2_ and ROS, thereby improving the hypoxic TME and mitigating hypoxia. The detection of ROS using the fluorescent probe DCFH_2_DA confirmed that InN@In_2_S_3_ can generate a substantial amount of ROS upon laser irradiation, leading to significant oxidative damage to the cells, a crucial requirement for PDT (Figure 10D). The *in vivo* findings further support these observations. Subsequently, an evaluation was conducted to determine the impact of oxidative stress on mitochondrial function, alterations in mitochondrial membrane potential, and the manifestation of superoxide generation, which indicated that under laser irradiation InN@In_2_S_3_ not only caused depolarization of the mitochondrial membrane but also induced cells to produce superoxide, proving oxidative stress damage. The traditional PDT or photothermal therapy is usually hindered by the low penetration depth of tissues. This study employed ICP-MS analysis of the major organs and tumor, as well as CT images, to establish the ability of InN@In_2_S_3_ to traverse the blood-brain barrier and accumulate within the tumor. Subsequently, the *in vivo* PDT efficacy of InN@In_2_S_3_ was assessed by monitoring tumor growth 2 weeks after laser irradiation (Figure 10E). Additionally, H&E staining and immunohistochemistry experiments were conducted to provide further evidence supporting the conclusion that the tumor inhibition and killing effects of InN@In_2_S_3_ are enhanced by 1,270 nm laser excitation (Liu et al., 2024).

Furthermore, the combination of photocatalytic O_2_ generation with other therapeutic strategies, such as chemotherapy or immunotherapy, has shown promising results (Shi et al., 2022). The increased O_2_ supply can enhance the efficacy of these treatments by overcoming tumor hypoxia and improving drug delivery. In order to achieve hypoxic tumor therapy, a novel Z-scheme heterostructured nanorods (NRs) consisting of bismuth sulfide (Bi_2_S_3_) and bismuth (Bi) are designed (Figures 10A, B). These nanorods facilitate the concurrent supply of O_2_ and the production of ROS in a spatiotemporally synchronized manner. This unique attribute of the Z-scheme heterostructure enhances hole separation in the valence band of Bi_2_S_3_ and electron separation in the conduction band of Bi, thereby promoting their robust redox capabilities. The vacancy within the valence band of Bi_2_S_3_ can react with H_2_O, providing O_2_ molecules for the electrons in the conduction band of Bi, resulting in the generation of ROS (Figure 10C). Computed tomography (CT) images demonstrated the effective tumor-targeting ability of LyP-1 on Bi_2_S_3_@Bi nanorods, while examination of HIF-1α expression in tumor tissues indicated successful alleviation of tumor hypoxia (Figures 10D–F). Consequently, the Bi_2_S_3_@Bi Z-scheme heterostructured nanorods exhibit significant potential for cancer diagnosis and therapy due to their capacity for CT imaging, enhanced photothermal performance triggered by NIR radiation, and mitigation of hypoxia limitations for PDT (Cheng et al., 2020).

In conclusion, photocatalytic O_2_ generation holds great potential for improving tumor PDT (Guo et al., 2017). By providing a local source of O_2_ within the TME, this technique can enhance the production of ROS and improve the therapeutic outcome of PDT. Further research and development in this field are needed to optimize the photocatalytic systems and explore their potential in clinical applications.

## 7 Conclusion and outlook

Here, we summarize the latest progress in the development of tumor-oxygenated nanomaterials for improving PDT’s therapeutic efficacy (Table 1). As part of the presentation, we discussed the multiple benefits of these biomimetic nanomaterials, including in-situ O_2_ generation, O_2_ delivery, tumor vasculature normalization, mitochondrial-respiration inhibition, and photocatalytic O_2_ generation. Compared to other materials, nano-enabled tumor oxygenation strategies improved tumor treatment outcomes by alleviating hypoxic TMEs.

**TABLE 1:** The PDT effect of different engineering tumor-oxygenated nanomaterials.

Agent	Cancer cell types	Effect	Therapeutic efficacy	Ref.
Ce6-CAT/PEGDA hydrogel	4T1 cells	30% (sO_2_ average in total tumor)	Enable multiple rounds of PDT	Meng et al. (2019)
PTX/ICG-NVs@Au@CAT	U14 cells	5.23% ± 2.16% (antitumor efficacy)	Increased the stability of PDT	He et al. (2019)
Au-Hb@PLT	HeLa and HEK cells	①The hypoxic tumors (2.367% ID g^−1^ tumor)	Directly deliver molecular oxygen and radiosensitizers to the tumor tissue	Xia et al. (2020)
		②Up to 90% of the mice were alive even at the end of the study (45 days)		
C@HPOC	Lung cancer	4.3 mg/L (O_2_ released rat)	Induced highly efficient tumor regression	Chen et al. (2018)
BLICP@O_2_	Hepatocellular Carcinoma	0.56 (^1^O_2_ quantum yield)	Photothermal heating triggers oxygen release	Zeng et al. (2023)
Endo@GOx-ER	4T1 cells	①the hypoxic tumors (uptake: 2.364% ID/g of tumor)	With a long-term tumor normoxic microenvironment and repeated RT for a long time	Huang et al. (2020)
		②90% (Survival rate of mice longer than 5 weeks)		
DEX-HAase	4T1 cells	①13.5% (the tumor sO_2_ average total)	Enhanced enzyme stability, reduced immunogenicity and prolonged blood half-life	Wang et al. (2019)
		②macrophage infiltration from 1.16% to 6.97%		
		③≈50% of mice survived over 50 days		
Ato-ICG-GNPs	Hela cells	66 mmol/L (ROS concentration)	Deeply into those truly hypoxic regions	Xia et al. (2019)
InN@In_2_S_3_	Glioblastoma	0.95 mg/L (O_2_ evolution)	With wide and strong near-infrared absorption and high electron-hole separation efficiency owing to its II heterojunction	Liu et al. (2024)
Bi_2_S_3_@Bi	4T1 cells	0.98 mg/L (O_2_ evolution)	With an efficient electron-hole separation ability and potent redox potentials	Cheng et al. (2020)

While some key issues remain unresolved, nano-enabled tumor oxygenation strategies have not yet been used to treat cancer in clinical trials as they still face challenges.

(1) Despite promising results in preclinical studies, many nano-enabled tumor oxygenation strategies have not demonstrated sufficient efficacy in clinical trials. The nanoparticles may not effectively deliver O_2_ to the tumor tissues or may not be able to overcome the complex TME (Sun et al., 2020).
(2) Some nano-enabled tumor oxygenation strategies may have safety concerns associated with them. For example, certain nanoparticles may induce toxicity or cause immune reactions in the body. These safety concerns need to be thoroughly evaluated before clinical application (Wang H. et al., 2020).
(3) The regulatory approval process for new therapies is rigorous and time-consuming. Nano-enabled tumor oxygenation strategies may face challenges in meeting the regulatory requirements for clinical use, including demonstrating safety and efficacy in large-scale clinical trials.
(4) The production and manufacturing of nanoparticles for clinical use can be expensive and challenging to scale up. This can limit the widespread adoption of nano-enabled tumor oxygenation strategies in clinical settings.
(5) There is a lack of standardized protocols for the use of nano-enabled tumor oxygenation strategies in clinical practice. This can hinder their widespread adoption and integration into existing treatment protocols.

Some possible solutions are listed below to address these challenges. Firstly, improving the tumor penetration of nano-enabled oxygenation agents can be achieved by modifying their surface properties (Zhang et al., 2019). This can be done by incorporating targeting ligands that specifically bind to tumor cells, enhancing their uptake and accumulation within the tumor tissue. Secondly, extending the circulation time of these agents can be achieved by modifying their size and surface charge (Mitchell et al., 2021). Increasing the size of the nanoparticles can prevent their rapid clearance by the immune system, while modifying their surface charge can reduce their interaction with blood components, thereby prolonging their circulation time. Lastly, enhancing the O_2_ release capability of these agents is crucial for effective tumor oxygenation (Ruan et al., 2021). This can be achieved by designing nanoparticles that respond to specific stimuli, such as low pH or high temperature, triggering the release of O_2_ within the TME.

The translation of nano-enabled tumor oxygenation strategies from preclinical studies to clinical applications presents several potential challenges and limitations. Such as, ensuring the safety of nanocarriers and oxygen delivery systems in humans is paramount. Developing strategies to enhance tumor targeting and minimize systemic toxicity is also crucial. Nanocarriers may face barriers such as poor tumor penetration, off-target accumulation, and clearance by the reticuloendothelial system. The cost of developing and manufacturing nano-enabled tumor oxygenation strategies may be high, potentially affecting their accessibility and affordability. Overcoming these challenges and limitations will require collaborative efforts among researchers, clinicians, regulatory authorities, and industry partners.

The field of nano-enabled tumor oxygenation strategies for PDT presents numerous promising research directions and opportunities for future advancements. (1) Enhanced oxygen delivery systems. Developing nanocarriers specifically engineered to transport oxygen more efficiently to tumor sites. Investigating the use of oxygen-generating nanoparticles or oxygen-releasing materials to locally increase oxygen concentration within the tumor microenvironment. Exploring strategies to improve the stability and controlled release of oxygen from nanocarriers to sustain tumor oxygenation during PDT treatment. (2) Targeted oxygen delivery. Designing targeted nanocarriers that can selectively deliver oxygen to tumor cells or tumor-associated vasculature, enhancing the specificity of PDT. Developing multifunctional nanocarriers that combine oxygen delivery with other therapeutic modalities, such as drug delivery or immunotherapy, for synergistic effects. (3) Nanoparticle design and engineering. Designing nanoparticles with tailored size, shape, and surface properties to optimize tumor penetration, cellular uptake, and PDT efficacy. Investigating the development of multifunctional nanoparticles that combine oxygen delivery with other PDT-enhancing functionalities, such as photosensitizer loading, targeting ligands, or controlled drug release. (4) Combination therapies. Exploring the combination of nano-enabled tumor oxygenation strategies with other PDT modalities, such as photodynamic immunotherapy, photothermal therapy, or photodynamic priming, to achieve synergistic effects and improve therapeutic outcomes. (5) Clinical translation and safety. Conducting comprehensive preclinical studies to evaluate the safety and efficacy of nano-enabled tumor oxygenation strategies in animal models of cancer. Investigating long-term effects and potential toxicity associated with nano-enabled tumor oxygenation strategies, ensuring the development of safe and sustainable therapeutic interventions. These research directions offer exciting opportunities to push the boundaries of nano-enabled tumor oxygenation strategies for PDT, leading to improved treatment outcomes and enhanced patient survival rates. Through continued research and innovation, the field has the potential to revolutionize PDT and provide new avenues for effective cancer treatment.

In conclusion, these advancements will pave the way for the clinical application of these strategies in the treatment of tumors. Future developments in cancer treatment will be influenced by biomimetic nanomaterials that are capable of oxygenating tumors and developing biomimetic nanomaterials.
